# Design, Synthesis, and Proof-of-Concept Bioassay of an Encapsulated mRNA for Human Growth Hormone

**DOI:** 10.3390/cimb48070647

**Published:** 2026-06-23

**Authors:** Carolina Rivera Santiago, Andrés Quintanar Stephano, Hugo A. Barrera Saldaña

**Affiliations:** 1 Laboratorios Columbia S.A. de C.V., Coyoacán, Ciudad de México 04000, Mexico; crivera@gcolumbia.com; 2 Laboratorio de Neurofisiología Básica, Universidad Autónoma de Aguascalientes, Ciudad Universitaria, Aguascalientes 20100, Mexico; andres.quintanar@edu.uaa.mx; 3 Vitagénesis S.A. de C.V./Innbiogem S.C., LANSEIDI-SECIHTI, Monterrey 64630, Nuevo León, Mexico; 4 Escuela de Medicina, Universidad Autónoma de Nuevo León, San Nicolás de los Garza 64460, Nuevo León, Mexico; 5 Escuela de Ciencias Biológicas, Universidad Autónoma de Nuevo León, San Nicolás de los Garza 66450, Nuevo León, Mexico

**Keywords:** human growth hormone, replacement therapy, mRNA-LNPs, hypophysectomized rats

## Abstract

Background: Human growth hormone (hGH) deficiency (GHD) is typically treated with daily injections of recombinant human growth hormone (rhGH), which do not fully replicate physiological secretion patterns. This study evaluates a novel approach using synthetic mRNA encoding hGH encapsulated in lipid nanoparticles (LNPs) and designated VTRC-01 to enable endogenous hormone production. Methods: VTRC-01 was administered intramuscularly to hypophysectomized (Hypox) prepubertal Wistar rats, and its efficacy was compared with rhGH. A cohort of healthy rats was included to assess anabolic effects and safety. Results: VTRC-01 stimulated longitudinal growth in both Hypox and healthy rats, achieving effects comparable to rhGH. Treatment induced a significant anabolic response that exceeded the basal growth rate of healthy controls. Conclusions: These findings provide proof-of-concept for hGH mRNA-based therapy as a promising alternative to rhGH. Further improvements in mRNA and LNP technologies are expected to enhance safe hormone production. These promising results underscore the potential of reprogramming via therapeutic mRNA the synthesis of key endocrine regulators (such as hGH) directly within the organism, offering for the first time a powerful pathway for the potential treatment for endocrine therapies targeting growth hormone deficiency.

## 1. Introduction

Human growth hormone (hGH) is a 191-aminoacidic-residue polypeptide synthesized and secreted in pulses by somatotropic cells in the anterior pituitary gland under complex neuroendocrine regulation. The axis constituted by hGH and its main mediator, insulin-like growth factor 1 (IGF-1), constitutes a central endocrine system in vertebrates, playing a pivotal role not only in regulating longitudinal growth but also energy metabolism and body composition. hGH acts directly mainly in adipose tissue and skeletal muscle and indirectly by inducing hepatic synthesis of IGF-I, which is mainly responsible for the growth of long bones [1].

The deficiency of hGH (GHD) represents a complex clinical entity with distinct implications across the lifespan. GHD in children, particularly the congenital and idiopathic forms, is manifested primarily as severe growth failure, delayed skeletal maturation, and altered body composition, often requiring early diagnosis and daily administration of recombinant hGH (rhGH) to optimize catch-up growth and achieve near-normal adult height. Collectively, these findings underscore that GHD is not merely a disorder of linear growth but a systemic condition with lifelong repercussions, necessitating individualized diagnostic strategies and enhancing the monitoring and optimization of the effectiveness of pharmacological interventions to mitigate adverse metabolic and musculoskeletal outcomes [2]. Current clinical indications for rhGH therapy encompass a broad spectrum of etiologies in both pediatric and adult populations. In pediatrics, the FDA has approved its use for growth hormone deficiency (GHD), Prader–Willi Syndrome, and Turner Syndrome, as well as for children born small for gestational age (SGA) who fail to manifest catch-up growth. Additional approvals include idiopathic short stature (ISS), SHOX gene haploinsufficiency, Noonan Syndrome, and growth failure associated with chronic renal insufficiency. In adults, GHD is associated with profound metabolic and functional consequences, including increased visceral adiposity, dyslipidemia, insulin resistance, reduced bone mineral density, impaired muscle strength, and diminished quality of life. These alterations contribute to elevated cardiovascular risk and potentially increased mortality in hypopituitary populations, although normalization of this risk with rhGH therapy requires further investigation [3,4]. Although rhGH therapy has maintained an exemplary track record of safety and efficacy, several studies have indicated that the treatment carries risks of long-term morbidities that remain incompletely understood [5]. Nevertheless, despite its well-established clinical efficacy, conventional therapy faces critical challenges regarding patient adherence, primarily due to the annoying daily subcutaneous injection regimen. This protracted treatment burden entails not only significant logistical and emotional complications for the patient and their family unit but also imposes a substantial long-term financial strain [6]. In response to these limitations, there is an imperative need to explore novel technologies that transcend conventional protein replacement therapy.

The clinical use of rhGH commenced in 1985 as a replacement for pituitary-derived hormone extracted from cadavers, which had been linked to the development of the devastating and deadly Creutzfeldt-Jakob disease [7]. The advent of the biosynthetic hormone represented a paradigm shift in pediatric endocrinology, providing a safe and virtually inexhaustible supply that facilitated the expansion of therapeutic indications from classical GHD to a broad spectrum of growth disorders.

Current biotech platforms rely on protein biosynthesis within heterologous expression systems, such as the conventional bacterial host Escherichia coli [8] or the more versatile yeast host Pichia pastoris [9]. The endogenous production of hGH through the reprogramming of the patient’s cellular machinery using synthetic hGH mRNA represents a cutting-edge alternative to the current genetic engineering-derived version of the drug. The therapeutic paradigm of synthetic mRNA has rapidly transitioned from a rapid-response vaccinology tool to corrective protein replacement therapies. However, the application of therapeutic mRNA to complex endocrine disorders remains virtually uncharted [10].

The objective of the present research was to perform, in an unprecedented way, the design, preparation, physico-chemical characterization, and initial biological efficacy evaluation of a novel nanoencapsulated formulation of hGH mRNA.

## 2. Materials and Methods

### 2.1. Design and Synthesis of VTRC-01

VTRC-01 corresponds to a synthetic mRNA coding for the main isoform (22 kDa; GenBank database accession number NM_000515.5) of hGH, encapsulated in LNPs. To generate it, a synthetic gene coding for it was manufactured (GenScript, Piscataway, NJ, USA) and cloned into the pD004cc vector using Age I and Mfe I restriction sites. The resulting synthetic gene included 5′ and 3′ untranslated regions (UTRs), transcription initiation and termination elements, and a polyadenylation tail, all organized to form an efficient transcription unit to act as a template for mRNA synthesis. Plasmid bacterial propagation was performed, and after isolation, it was characterized to verify its identity, purity, and topological conformation. The linearized plasmid was subsequently used as a template in a laboratory-scale in vitro transcription (IVT). During the reaction, uridine was fully replaced with N1-methylpseudouridine (Ψ) to reduce immunogenicity and improve its translation. Purified mRNA was characterized by quality control, ensuring high purity and molecular integrity. mRNA integrity and identity were verified using automated capillary electrophoresis (TapeStation, Agilent Technologies, Santa Clara, CA, USA). Additionally, mRNA concentration and purity were determined by assessing the A260/A280 absorbance ratio using a DeNovix DS-11 spectrophotometer (DeNovix Inc., Wilmington, DE, USA). mRNA encapsulation was performed using a high-impact microfluidic mixing technique. For the formation of the LNPs, a biocompatible ionizable lipid (SM-102) was used as the primary component of the organic phase. Cholesterol, 1,2-distearoyl-sn-glycero-3-phosphocholine, and DMG-PEG 2000 were also components of the formulation. The lipid mixture was combined with a sodium acetate buffer (50 mM, pH 4.5) containing the mRNA at a 3:4 volume ratio (aqueous: organic) using a microfluidic mixer (Precision NanoSystems, Vancouver, BC, Canada). The resulting LNPs were dialyzed against 12 mM Tris-HCl in 10% *w*/*v* sucrose and sterilized by passage through a 0.22 µm filter. The final particles exhibited a mean hydrodynamic diameter of 92.45 ± 3.89 nm with an RNA encapsulation efficiency exceeding 70%. Template sequence optimization, mRNA synthesis by IVT, and encapsulation were performed at the RNA Core service of the Houston Methodist Research Institute (Houston, TX, USA).

### 2.2. A Murine Model to Test Growth Promotion Activity

It is well established that hGH displays biological activity across the evolutionary scale, including in fish, whereas the inverse does not occur as animal-derived GH variants lack activity in humans. Therefore, we selected hypophysectomized (Hypox) rats as the surrogate model to assess the growth-promoting activity of the hormone.

Prepubertal female Wistar rats (21 days old) were bred at the Animal Facility of the Autonomous University of Aguascalientes (Aguascalientes, México). All experimental procedures were conducted in strict accordance with the guidelines established by the Institutional Animal Ethics and Welfare Committee (Protocol No. CEADI/UAA/0025/18; CEADI-UAA/03/2026). For their preparedness, the animals underwent an acclimatization period of 5–7 days under controlled environmental conditions, including a 12:12 h light/dark cycle and a constant temperature of 24 ± 2 °C. Food and water were provided ad libitum.

Hypophysectomy was performed on rats aged 26–28 days under anesthesia with isoflurane (3–4% at 50 mL/min; Kent Scientific SomnoSuite Low-FloTXAnesthesia System (Kent Scientific Corp., Torrington, CT, USA), intubated, and placed in a supine position. Using a stereomicroscope-assisted transethmoidal approach, a 2–3 mm craniotomy was performed at the occipito-sphenoidal junction. The hypophyseal capsule was incised, and the pituitary gland was removed via suction. Postoperatively, animals received a single intramuscular dose of antibiotics and analgesics and were monitored in a recovery chamber (26 ± 2 °C) with supplemental oxygen. Long-term maintenance included an enriched diet and 5% glucose solution to manage transient diabetes insipidus. Successful hypophysectomy was verified by monitoring the severe growth retardation (in body weight and nose-to-anus length) daily for 12 days. Inclusion criteria for the experimental phase required a body weight gain of less than 6 g and a linear growth of less than 1 cm.

### 2.3. Experimental Design for Evaluating the Growth-Promoting Activity of VTRC-01

The animals were assigned to two primary group types: intact control (IC) subjects and hypophysectomized (Hypox; surgically intervened) subjects. In turn, animals in the groups were assigned to subgroups based on the treatment regimens [hGH mRNA (the experimental new drug) versus rhGH (the reference drug, HHT^®^ 4 IU, Biosidus SA. Buenos Aires, Argentina) acquired at a local pharmacy] as detailed in Table 1. The mRNA dose administered to each animal was adjusted based on the concentration of the available aliquots, ranging from 8 to 20 µg, while maintaining a constant injection final volume of 80 µL. Growth monitoring was assessed by measuring body weight and nose-to-tail length on days 4, 7, 10, 12, 33, 41, and 48 post-hypophysectomy. Necropsy was performed on day 48, at which time a macroscopic inspection of the Sella turcica was conducted to confirm the absence of pituitary remnants, thereby providing final validation of the surgical success and thus of the results. Furthermore, this macroscopic inspection included basic morphological observations and weight measurements of relevant internal organs.

### 2.4. Treatment Administration

The administration of VTRC-01 or rhGH was performed via intramuscular (IM) injection into the posterior aspect of the thigh (targeting the gluteus maximus muscle). Treatments were initiated 17 days post-hypophysectomy, with daily intramuscular injections alternating between the left and right thighs. On the day of the experiment, LNP aliquots containing hGH mRNA (stored at −80 °C following synthesis) were thawed at room temperature (5–10 min, depending on volume) and then maintained at 4 °C before administration. For doses requiring dilution, a buffer solution consisting of 10% (*w*/*v*) sucrose and 12 mM Tris-HCl (pH 7.4) was used. The rhGH was reconstituted according to the manufacturer’s instructions and further diluted with saline solution to administer a daily dose of 5 µg.

### 2.5. Statistical Analysis

To evaluate the effect of the different treatment regimens across subgroups, two distinct statistical strategies were implemented: a Linear Mixed Effects Model (LMM) and a one-way analysis of variance (ANOVA), followed by Tukey’s post hoc test for multiple comparisons. The results were presented as mean ± standard error of the mean (SEM). Statistical significance was set at *p* < 0.05. Statistical analyses were performed using the jamovi software, version 2.5 (The jamovi project, 2024). This program was selected due to its integration of R-based statistical engines, providing a robust environment for the execution of LMM. Time was utilized as a continuous covariate within the LMM framework to account for irregular intervals. The ANOVA analyses were conducted using GraphPad Prism software (version 9.5.1).

## 3. Results

### 3.1. Synthesis and Characterization of VTRC-01 mRNA

#### 3.1.1. Synthetic DNA and Cloning

**Figure 1:**
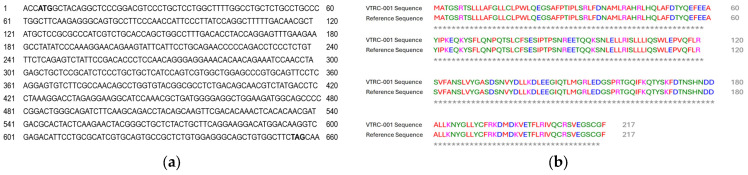
Sequences of the VTRC-01 template and encoded protein. (**a**). The primary structure of the mRNA encoding for hGH, shown as its DNA version. The start (ATG) and stop (TAG) codons are highlighted in bold, flanking the translatable codons for the protein of interest. (**b**). The amino acid sequence was conceptually translated from the designated start and stop codons. The asterisks below the alignment indicate full identity between both sequences. This sequence corresponds to the reference human pituitary growth hormone precursor, as retrieved from the Swiss-Prot database (Accession Number: P01241).

**Figure 2:**
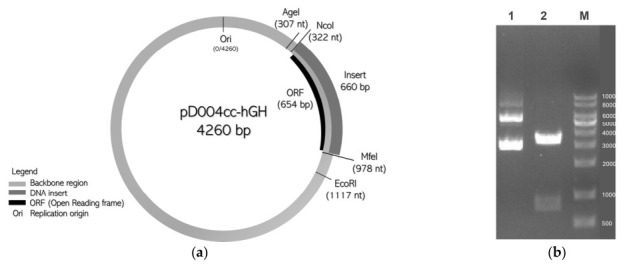
Verification of recombinant plasmid integrity. (**a**). Restriction map. (**b**). Gel electrophoresis. Lane 1 displays the undigested plasmid in its native state, whose supercoiled isoform predominates, confirming the integrity and high purity of DNA extraction. Lane 2 shows the results of the double digestion using Nco I and Eco RI restriction enzymes; the release of the lower molecular weight band verifies the presence and integrity of the gene of interest inserted into the vector. Finally, Lane M corresponds to the molecular weight marker (DNA ladder in nucleotides), used to estimate the size of the resulting bands.

Following synthesis, the *hGH-1* gene exonic sequence was inserted into the pD004cc plasmid. This sequence, retrieved from GenBank, comprises 654 base pairs (bp) and encodes a total of 217 amino acids (aa), corresponding to 191 to the mature hormone and 26 aa to its signal peptide. In addition, the sequence was flanked by a Kozak sequence at the 5’ end, and a termination codon followed by a poly(A) tail at the 3’ end. Figure 1a illustrates the nucleotide sequence of the template used for the in vitro transcription (IVT), and Figure 1b depicts the amino acid sequence encoded in it; the identity of both sequences was verified through in silico analyses. The restriction map of the recombinant plasmid harboring the synthetic gene used as the template for the synthesis of the hGH mRNA is illustrated in Figure 2a. After bacterial propagation and purification, the plasmid was characterized to confirm its expected molecular weight, integrity, and identity (Figure 2b). Purity was assessed via the A260/A280 ratio (1.92), and the total yield was ≥4 µg, satisfying the established specifications (1.8–2.0 and 4 µg, respectively).

#### 3.1.2. Characterization of mRNA Following Encapsulation

**Figure 3:**
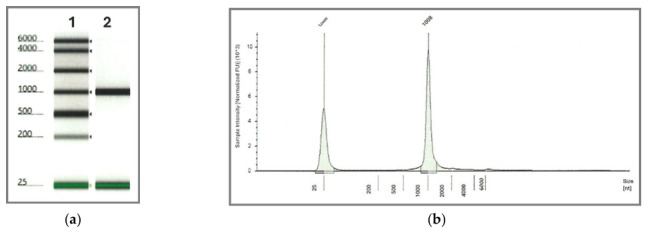
mRNA integrity assessment following encapsulation. (**a**) Automated electrophoresis gel image. Lane 1: Molecular weight marker in nucleotides (nt); Lane 2: Purified mRNA transcript showing a single, sharp band at approximately 1000 nt, indicating high purity and absence of degradation. (**b**) Representative electropherogram from TapeStation analysis. The sharp peak at 1008 nt confirms the expected size and structural integrity of the mRNA. The 25-nt position band represents a method of control.

The LNPs were characterized based on the following physicochemical properties: 1. Size (nm): Refers to the particle diameter; the target range is 55 nm to 150 nm. 2. Polydispersity index (PDI, arbitrary units): A value indicating the size distribution of the nanoparticles. Values closer to 0 represent a monodisperse (uniform) population, while values closer to 1 indicate higher heterogeneity. A PDI < 0.22 is considered acceptable. And 3. zeta potential (ZP, mV): Represents the surface charge of the nanoparticles. A ZP < −1 mV is considered acceptable for these formulations. As shown in Table 2, the quality criteria are within acceptable limits. The synthesized material (30 mg divided into 10 batches of 3 mg on average each) exhibited optimal quality based on three key parameters: First, structural integrity was maintained, demonstrating that neither the LNP encapsulation process nor the subsequent release for analysis damaged the mRNA strand. Second, sequence identity was confirmed, as the peak at 1018 nucleotides (nt) aligned with the expected transcript size (Figure 3a). And third, the absence of contaminants was verified by the lack of secondary peaks (Figure 3b).

### 3.2. The Hypophysectomy Experimental Animal Model

**Figure 4:**
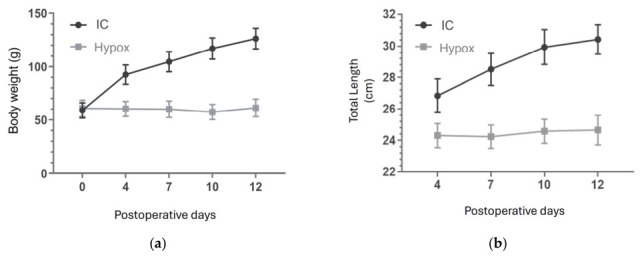
Somatic parameters of control and experimental animals. (**a**). Body weight curve post-hypophysectomy. (**b**). Nose-to-tail length curves. The data are expressed as the mean ± SEM. Intact control (IC); n = 20; hypophysectomized (Hypox); n = 22.

The body weight and total length (nose-to-tail) gain curves prior to treatment of post-hypophysectomy animals in the different groups are shown in Figure 4. A significant retardation in body weight and longitudinal growth gains was observed in prepubertal rats subjected to hypophysectomy, confirming the essential role of the pituitary gland (and thus of its own growth hormone) in somatic development and metabolism. At this stage, group assignment remained preliminary, as the presence of residual pituitary tissue was further assessed during post-mortem inspection. Regarding surgical outcomes, 22 of 29 rats survived the procedure, yielding a 75.8% survival rate.

#### Survival Rate

The analysis of survival rates at the conclusion of the treatment period revealed the consequences of hypophysectomy and reflected the learning curve in their post-surgery care. While the Hypox animals had a rate of only 54% (12 out of 22 subjects), the intact animals had 100%. Again, reflecting the intrinsic risk associated with the surgical intervention and the physiological fragility of this endocrine model. As indicated in Table 3, it is essential to consider that, in addition to mortality, the sample size (n) was further reduced by the exclusion of subjects identified with pituitary remnants. Regarding mortality, the suspected causes include the deficient temperature control of the animal facility, nutritional energy deficiencies, high drug administration volume, the learning curve of the surgical procedure, and lack of rigorous control of the pH of the diluent of VTRC-01. Consistent with the outcomes of the Hypox VTRC-01 20 µg group, all animals succumbed to the uncontrolled instability of the mRNA formulation vehicle.

### 3.3. Comparative Evaluation of mRNA and rhGH Administrations

**Figure 5:**
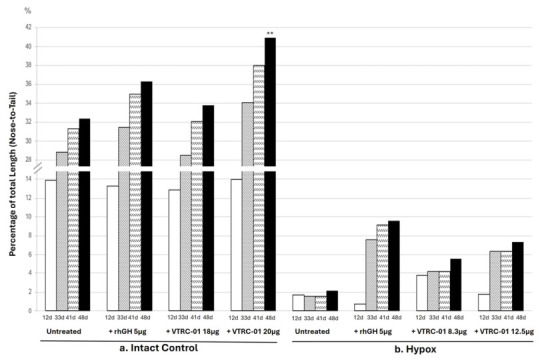
Effect of treatments on longitudinal body growth. *Y*-axis: Percentage of nose-to-tail growth. *X*-axis: Subgroups of treatments and follow-up at 12, 33, 41, and 48 days (d). (**a**) Intact control group. (**b**) hypophysectomized group (Hypox). ** *p* < 0.01.

The effect of the administration of the medications (rhGH acting as the reference drug and the hGH mRNA as the experimental one) on the size of intact and surgically intervened animals is illustrated in Figure 5. In the case of intact control (IC) animals, the maximum extra size gain was seen in the rats that received the highest mRNA dose (20 µg) on day 48, compared to those receiving rhGH (5 µg) and even more dramatically to the untreated IC animals (Figure 5a). On the other hand, Hypox animals that received no treatment showed the lowest increase in total length, the highest when treated with rhGH (5 µg), and a comparable gain when treated with hGH mRNA at both doses (8.3 and 12.5 µg) (Figure 5b). If these gains in nose-to-tail lengths are expressed as a percentage and compared to the size of animals on the day equivalent to the date of the surgery (taken from the Hypox animals), then in the first category of animals (IC) without treatment a growth rate of 32.15% was shown (from 26.94.72 ± 0.76 to 35.60 ± 0.58 cm). In comparison, the administration of rhGH (5 µg) induced a stimulatory effect on nose-to-tail longitudinal growth of 36.18% (from 26.90 ± 0.58 to 36.63 ± 0.39 cm), representing an increase of 4.04%. Notably, the subgroup receiving hGH mRNA (20 µg) exhibited an even greater stimulatory effect on body growth (40.80%, *p* < 0.01; from 26.35 ± 0.43 to 37.10 ± 0.46 cm). It is important to mention that no apparent adverse effects were observed in these intact animals following mRNA administration; instead, the treatment resulted in growth stimulation comparable to that induced by rhGH. Although the high mortality rate among Hypox subjects precludes a formal statistical analysis for the effects of treatments on the animals’ size, the data reflect that hGH mRNA effectively stimulates growth. Specifically, Hypox animals treated with mRNA at doses of 8.3 and 12.5 µg exhibited an average body growth gain three times greater than that of untreated Hypox subjects. Growth increased from 24.83 ± 0.12 to 26.20 ± 0.10 cm and from 23.70 ± 0.29 to 25.43 ± 0.23 cm for the respective dose groups, compared to a marginal increase from 25.75 ± 0.05 to 26.30 ± 0.10 cm in the untreated control.

#### Growth Dynamics Analysis by LMM

Before the experiment reported in this study, several similar pilot assays were conducted to optimize the experimental model (see Tables S1–S4, Supplementary Materials). These datasets were consolidated solely to perform a robust analysis of the experimental design. As a primary result, the LMM allowed the visualization of two approaches to evaluate the interaction between treatments and subject conditions (Hypox or IC), as detailed in Table 4. Approach A includes exclusively subjects with ‘no pituitary remnants’ (n = 59), thereby eliminating any biological confounding factors. The resulting *p*-value (*p* = 0.021) confirms that the treatment is effective independently, even in the most conservative scenario. In Approach B, all subjects were included (n = 89) by statistically adjusting for pituitary remnants, which increased the sample size. The *p*-value (*p* = 0.002), reflecting significantly higher statistical power, demonstrates that the model can correct the noise caused by incomplete surgery. The second relevant finding notably indicates that, within the IC group, the mRNA 20 µg treatment exhibited an anabolic effect that was numerically greater than that of rhGH. While in the Hypox group, although the biological activity elicited by the mRNA was confirmed, it was not statistically superior to that of the hGH reference standard.

- IC Group.

The LMM demonstrated high predictive power, with a coefficient of determination (R^2^) of 0.897. This indicates that the model accounts for 89.7% of the total variance in longitudinal growth, confirming a robust fit between the experimental data and the theoretical framework.

The interaction effect between time and mRNA dosage in comparison with the reference standard, rhGH, in the IC group is shown in Table 5.

- Hypox Group.

When analyzing the Hypox subjects exclusively, the goodness of fit of the model increases to 93.9% (conditional R^2^ = 0.939). The interaction effect between time and mRNA dosage in comparison with the reference standard, rhGH, in the Hypox group, is shown in Table 6.

## 4. Discussion

The unprecedented success of mRNA vaccines during the COVID-19 pandemic demonstrated logistical superiority in scalability and manufacturing speed, validating this technology as the successor to microorganism-based biotechnological methods [11]. As gene therapy, mRNA operates exclusively within the cytoplasm, eliminating the risk of genomic structural alterations. This mechanism simplifies protein synthesis, bypassing the nucleus: LNPs protect the fragile cargo from extracellular RNases and facilitate endosomal release directly to the ribosomes. Beyond protection, the tuning capability of LNPs allows for precise adjustments to optimize cellular uptake, enhance shelf life, and even enable tissue-specific targeting (tLNP). While challenges in expression duration persist, LNP modulation positions mRNA as a versatile platform for next-generation biotherapeutics designed to maximize efficacy and minimize side effects [12,13,14,15].

The therapeutic landscape of mRNA now extends far beyond vaccinology. In cardiovascular medicine, modified mRNA promotes myocardial regeneration and angiogenesis after infarction [16], while in oncology, it enables personalized vaccines encoding tumor-specific antigens (TSAs) to mobilize polyspecific immunity. However, its potential in metabolism and growth remains largely untapped. The use of mRNA as a therapeutic, a long-sought desire, is now revolutionizing the pharma landscape as a potentially superior biotechnology alternative to recombinant DNA (rDNA) and even to hybridoma platforms for the manufacturing of biotech drugs [17].

Recombinant hGH was among the first successes of rDNA in the modern biotechnology era [8]. Two main challenges remain, especially in pediatric endocrinology, to fully harness its undoubted benefits. The first challenge is optimizing the dosing interval, as the burden of current daily injections entails not only significant logistical and emotional complications for the patient and their family unit but also imposes a substantial long-term financial strain. The second challenge involves replacing injections with a less invasive delivery method. While attempts are in progress to address the first of these challenges by, for example, PEGylation of the hormone to extend its half-life [18], therapeutic mRNA offers better posology given that injected mRNA could last several days, yielding its encoded protein [19]. On the other hand, drug delivery advancements in medical devices, such as microneedle patches, leverage LNP administration to facilitate minimally invasive delivery [20]. However, any such proclamation postulating the potentiality of this promising technology in such terms needs to start with a proof-of-concept to show that indeed the mRNA alternative to the protein is at least as safe and efficacious as this latter.

Here, by using mRNA coding for hGH (VTRC-01) to stimulate body growth and length in an animal-relevant model (hypophysectomized prepubertal female Wistar rat), proof-of-concept is provided for the first time. Achieving this objective required addressing significant technical challenges, ranging from the design, synthesis, and formulation of VTRC-01 mRNA to the optimization of surgical procedures to ensure Hypox animals’ survival and the rigorous handling of the mRNA drug for experimental administration.

The procurement of high-quality experimental materials constituted the primary critical pillar for the success of this study. The evaluation of identity, purity, and yield parameters for the plasmid harboring the synthetic DNA template yielded not only satisfactory results but also strictly aligned with international regulatory requirements [21,22]. As established in the literature, a contaminant-free DNA template with a predominantly supercoiled topology is vital to minimize transcriptional errors and ensure high fidelity during the in vitro transcription (IVT) process [23]. Once the quality of the DNA template was ensured and it was in its linearized form, the synthesis and subsequent encapsulation of the purified and intact VTRC-01 mRNA yielded a formulation with optimal physicochemical properties. Compliance with particle size, polydispersity index (PDI), and zeta potential parameters, coupled with the analytical confirmation of integrity and identity, validated experimental robustness. According to Webb et al. (2025), the stringent evaluation of these Critical Quality Attributes (CQAs) ensures that the mRNA can evade enzymatic degradation and effectively reach the cellular machinery [24]. The design and manufacture of mRNA VTRC-01 demonstrate a close correlation with the industrial and quality standards described in the literature. These alignments with international CQAs, including transcript integrity and impurity control, validate the robustness of the VTRC-01 platform for future applications in the development of advanced therapies [25]. It is important to emphasize that chemical modifications (e.g., uridine replaced with N1-methylpseudoridine) and nanoparticle-mediated delivery have contributed significantly to improving mRNA stability, reducing intrinsic immunogenicity, protecting the molecule from enzymatic degradation, and enhancing both cellular uptake and endosomal escape [26].

The implementation of the hypophysectomized rat model represented a significant technical challenge, requiring the simultaneous control of multiple physiological variables. The validation of the procedure was based, in the first instance, on weight monitoring during the stabilization (run-in) phase; the absence of significant weight gain in the subjects before treatment confirmed the success of the surgery (see Figure 4). This validation, complemented by the post-mortem inspection of the Sella turcica to rule out pituitary remnants, guaranteed that the growth observed after the administration of VTRC-01 or rhGH is attributable exclusively to therapy and not to residual endogenous production. In contrast to contemporary genetic models, surgical hypophysectomy is considered an uncommon mechanical induction model, as it does not depend on genomic manipulations but rather on the physical ablation of the gland [27]. Being the hypophysectomized rat, the chosen ‘gold standard’ bioassay for evaluating the biological efficacy of VTRC-01, its viability depends on rigorous control of homeostasis. We identified that mortality rates are primarily associated with three critical factors converging specifically in the hypophysectomy: (1) metabolic and hemodynamic instability derived from acute panhypopituitarism (specifically the loss of ACTH and TSH signaling), which induces a state of systemic frailty treatable through electrolytic and thermal support [28]; (2) the narrow therapeutic window of anesthetic management in invasive procedures, which increases the risk of hypothermia and respiratory failure [29]; and (3) intraoperative hemorrhage due to the rupture of venous sinuses at the cranial base, the leading cause of acute mortality [30]. While the literature reports survival rates exceeding 90% in highly optimized protocols [31], the low 46% success rate observed in this study is largely attributable to limitations in postoperative care management and the technical proficiency required for the procedure. To mitigate the impact of this survival rate on the robustness of the data, an LMM was employed. This statistical tool is superior to traditional analysis of variance methods, as it allows for the inclusion of subjects with incomplete data (premature deaths), thereby avoiding selection bias and providing unbiased estimates of the therapeutic effects [32]. Finally, the high goodness of fit obtained (R^2^ = 0.897 and 0.93) suggests that, despite the intrinsic frailty of the model, the controlled factors (treatment and time) explain a substantial portion of the observed biological response, thereby supporting the evaluation of the biological activity of the new VTRC-01 drug candidate. However, while this statistical framework mitigates experimental noise, it serves as an analytical aid rather than a solution to the inherent biological and structural limitations of the animal model.

Following treatment administration, our results suggest that both rhGH and VTRC-01 exerted a biological effect. The application of an LMM was advantageous in filtering experimental noise and the intrinsic variability of the model, providing a statistical robustness that addresses the limitations of conventional ANOVA analysis when handling longitudinal data. Both biotherapeutics appeared to stimulate longitudinal growth in somewhat comparable magnitudes in both the Hypox rat model and the intact control (IC) subjects. These findings are consistent with the literature documenting growth retardation in models with pituitary gland deficiency and the restorative capacity of rhGH therapy [33]. It is imperative to highlight that, to the best of our knowledge, this study represents the first report of growth induction mediated by the expression of an exogenous hGH mRNA. Since longitudinal growth strictly depends on the development of long bones at the epiphyseal plate, we hypothesize that this stimulation is attributable to the indirect action of the hGH encoded by VTRC-01. Given the nature of the Hypox model utilized, quantifying the serum concentrations of circulating hGH and their hepatic mediator IGF-1, along with additional molecular analyses, is a pending assignment for future studies to provide definitive, direct biochemical evidence of success in vivo mRNA translation and target pathway activation. This physiological mechanism is proposed to involve the induction of hepatic IGF-1 synthesis, the fundamental systemic mediator of the somatogenic effects of growth hormones [34]. The observation of these indirect actions further suggests the therapeutic potential of employing mRNA platforms that directly encode this mediator in future investigations. While the initial study design aimed to evaluate a dose-dependent response, the procedural limitations inherent in establishing, for the first time, the highly fragile rat Hypox model, as well as constraints associated with aliquot concentration and the need to avoid multiple thawing to prevent mRNA degradation, restricted our ability to assess the full dose spectrum. Future studies will require an optimized Hypox model, animal group sample sizes adequate for rigorous formal statistical analysis, and compliance with rigorous laboratory quality standards. Furthermore, to fully elucidate the comparative pharmacodynamics and determine the exact relative potency of the therapeutic mRNA alternative, future investigations will be designed using molar-equivalent and identical dosing regimens between VTRC-01 and rhGH.

**Figure 6:**
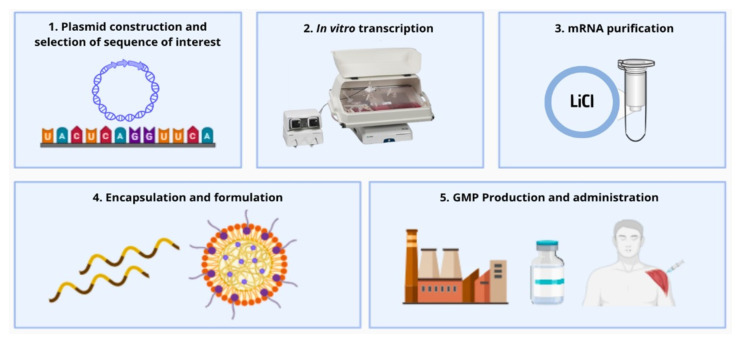
Workflow for the production and administration of mRNA-LNP-based therapeutics. The process comprises five critical stages: 1. Plasmid construction: design and selection of the genetic sequence of interest encoding the therapeutic protein. 2. In vitro transcription (IVT): enzymatic synthesis of mRNA using a linearized DNA template. 3. mRNA purification: removal of byproducts and reagents through LiCl precipitation or chromatographic methods. 4. Encapsulation and formulation: self-assembly of mRNA within lipid nanoparticles (LNPs) to ensure stability and cellular delivery. 5. GMP Production and administration: scaling under Good Manufacturing Practice (GMP) standards for subsequent clinical application via injection. The schematic representation was created with Canva IA 2.0 (Canva Pty Ltd. Sydney, Australia, 2026).

From a safety standpoint, mRNA therapy mitigates critical metabolic risks. While conventional rhGH induces supraphysiological peaks associated with insulin resistance [35], mRNA-LNPs enable more stable and controlled kinetics of expression; in other words, this platform facilitates prolonged protein production rather than the acute systemic exposure typically associated with recombinant protein boluses [36], thereby reducing diabetogenic stress. Additionally, the versatility of LNPs could allow for the co-encapsulation of multiple mRNA sequences (e.g., hGH and IGF-1) within a single nanoparticle [37]. This capability is vital for managing complex cases of hGH deficiency with peripheral insensitivity, enabling precision medicine through dynamic dose adjustments tailored to the patient’s developmental stage. Finally, in vitro transcription (IVT) provides unprecedented scalability, and since the manufacturing process is sequence-independent, the transition to manufacturing different therapeutic proteins simply requires the modification of the nucleotide sequence [38]. Compared to recombinant DNA technology, which relies heavily on complex bio-industrial manufacturing infrastructure, the infrastructure required for the fabrication of therapeutic mRNA is significantly simpler (Figure 6).

Nevertheless, the adoption of mRNA technology to replace current biotech drugs, here exemplified by the case of rhGH, to innovate hGH replacement therapy, still faces significant technical hurdles. The intrinsic instability of mRNA and the propensity of LNPs for aggregation still necessitate ultra-low temperature storage (−20 °C to −80 °C), complicating logistics in resource-limited settings [39]. Regarding immunogenicity risk, despite the incorporation of modified nucleotides such as pseudo-uridine, ionizable lipids may trigger the innate immune system or induce anti-PEG antibody production, potentially compromising efficacy during chronic administration [40]. Lastly, the current cost of GMP-grade raw materials and intellectual property constraints regarding LNPs represent economic barriers that currently exceed the production costs of traditional bacterial recombinant biotherapeutic platforms [41].

### Study Limitations and Future Perspectives

Despite the favorable results obtained, several limitations of this study must be acknowledged. First, the complexity of the hypophysectomy surgical technique limited the sample size due to the learning curve and the inherent intraoperative mortality of the model. Another critical obstacle involved the detection of pituitary remnants, which could only be evidenced through macroscopic or histological inspection following the necropsy of the subjects at the end of the experimental period. The presence of such remnants nullifies the total hypophysectomy condition, representing a retrospective challenge for the integrity of the study groups. Although statistical power was barely sufficient to infer significant differences, increasing the number of subjects while reducing the risk of hypophysectomy-associated mortality would further strengthen the robustness of the findings. Another limitation of the present study relates to its scope since it was limited to a short-term evaluation. Future investigations should address the impact of prolonged administration regimens, hopefully with less dosing frequency and delivery by sustained-release devices. While the rescue of longitudinal growth and organ development in the treated Hypox animals provides evidence of mRNA translation, the absence of circulating hGH/IGF-1 quantification and microstructural tissue analyses (such as growth plate histology, hepatic biomarkers, and cytokine panels) remains a limitation in resolving the precise pharmacokinetic profile of this hGH mRNA alternative to rhGH. Longitudinal growth outcomes are reliable, downstream indicators of somatotropic axis activation; however, they reflect the cumulative biological effect rather than acute protein expression kinetics. Future studies will integrate time-course serum protein quantification and gene expression profiling within target tissues to fully elucidate the systemic signaling cascade and optimize therapeutic dosing regimens. Furthermore, while phenotypic outcomes and macroscopic inspection of internal relevant organs validate initial therapeutic efficacy and safety of VTRC-01, to further strengthen these initial conclusions, particularly regarding safety, histological examination of biopsies is in progress. In this context, a preliminary hepatic histopathological assessment was performed, revealing that subjects in the VTRC-01-treated Hypox groups (8.3 µL and 12.5 µL doses) exhibited no observable hepatocellular damage. However, the VTRC-01-treated IC group, receiving an alternate dose of 20 µg over 20 days, displayed significant changes in hepatic cytoarchitecture, including microvesicular steatosis, nuclear pleomorphism, the presence of hepatocytes arranged in rosette-like structures, an increased number of binucleated hepatocytes, and vascular congestion. Collectively, these findings are consistent with an ongoing hepatic regenerative response. Overall, the treatment was well tolerated, with no significant body weight loss or adverse clinical signs observed during the duration of the study, suggesting that these preliminary findings do not represent overt systemic toxicity, but rather homeostatic adaptations described in the literature for high-dose lipid vehicle clearance. Nonetheless, it is imperative to conduct a more robust and comprehensive safety and toxicological profile to corroborate these initial approximations. It is a priority to optimize biopharmaceutical formulation to reduce the potential immunogenicity of the lipid nanoparticles (LNPs) and to refine storage conditions to mitigate the thermal instability of the mRNA. To address these analytical challenges and robustly validate the specific somatogenic effects of VTRC-01, subsequent studies will systematically incorporate the following efficacy and safety biomarkers:

(a) Epiphyseal growth plate thickness metrics to microscopically quantify chondrocyte proliferation.
(b) Definitive longitudinal measurement of the tibia bone.
(c) Molecular analysis of VTRC-01 half-life and duration of protein expression across target and off-target relevant organs in both Hypox and IC subjects.
(d) Rigorous biochemical and histological validation, including hepatic transaminases (ALT, AST) and comprehensive cytokine panels, to fully characterize the systemic toxicity and immunogenicity profiles.

These analytical challenges are considered within a new technological landscape: the recent emergence of promising new mRNA therapeutic platforms, such as circular and self-amplifying mRNA, in addition to new knowledge on mRNA coding sequence optimization [40].

Within the framework of disruptive advancements, mRNA platforms exert a significant potential impact on administration methods. In the future, for example, drug delivery via intradermal microneedles and, even more, through slow-release, refillable implantable devices, could enable a minimally invasive delivery of this new class of mRNA-based medications [39]. Finally, beyond the application of the mRNA-based drug successfully evaluated here in the reprogramming of growth and metabolism in pediatric patients, there is a renewed interest in exploring its benefits in the wellness of older individuals in general and in early-onset GH deficiency of the adult, in particular [42].

## 5. Conclusions

This study established, for the first time, proof-of-concept for a disruptive, innovative alternative to the administration of a biosynthetic version of hGH. Our findings demonstrate the biological activity of our new candidate mRNA-based drug comparable to that of this version of the hormone, thereby contributing to the promise of therapeutic mRNA technology as an alternative to recombinant biotherapeutics. These promising results underscore the potential of reprogramming, via therapeutic mRNA, the synthesis of key endocrine regulators (such as hGH) directly within the organism, offering a powerful pathway for the potential treatment of endocrine therapies targeting growth hormone deficiency.

## 6. Patents

The authors declare that a patent application has been filed related to the technology described in this study.

## Figures and Tables

**Table 1:** Description of treatment groups.

Group	Subgroup	Dose ^2^	Rats (n)	Injections (Days)	Frequency
IC ^1^	Untreated	N/A	5	N/A	N/A
IC	+rhGH	5 μg	6	10	Alternating
IC	+VTRC-01	18 μg	4	20	Daily
IC	+VTRC-01	20 μg	4	10	Daily
Hypox	Untreated	N/A	2	N/A	Daily
Hypox	+rhGH	5 μg	4	20	Daily
Hypox	+VTRC-01	8.3 μg	4	10	Daily
Hypox	+VTRC-01	12.5 μg	4	10	Alternating
Hypox	+VTRC-01	18 μg	4	20	Daily
Hypox	+VTRC-01	20 μg	4	10	Daily

^1^ Internal or intact control animals. ^2^ The expressed dose was administered per day. Abbreviations: µg, micrograms; n, number of subjects; and N/A, non-applicable.

**Table 2:** VTRC-01 mRNA characterization.

Parameter	Mean ^1^	±SD ^1^
Size (nm)	92.45	0.57
PDI (a. u.)	0.17	3.89
ZP (mV)	−6.78	0.02

^1^ The mean and ±SD were calculated using data from the 10 manufactured mRNA batches. Abbreviations: PDI: polydispersity index; ZP: zeta potential; nm: nanometers; a. u.: arbitrary units; mV: millivolts; and ±SD: standard deviation.

**Table 3:** Surviving animals in each experimental subgroup.

Group	Subgroup	Dose ^2^	n1	n2	% Survival
IC ^1^	Untreated	N/A	5	5	100
IC	+rhGH	5 μg	6	6	100
IC	+VTRC-01	18 μg	4	4	100
IC	+VTRC-01	20 μg	4	4	100
Hypox	Untreated	N/A	2	2	100
Hypox	+rhGH	5 μg	4	1	25
Hypox	+VTRC-01	8.3 μg	4	4 ^3^	100
Hypox	+VTRC-01	12.5 μg	4	3	75
Hypox	+VTRC-01	18 μg	4	0	0
Hypox	+VTRC-01	20 μg	4	2 ^4^	50

^1^ Internal control. ^2^ The expressed dose was administered per day. ^3^ One subject in this group was excluded from the analysis. ^4^ The two surviving subjects in this group were excluded, both (^3^ and ^4^), due to the post-mortem detection of pituitary remnants during the necropsy following euthanasia. Abbreviations: µg, micrograms; n1, initial number of subjects; n2, number of surviving subjects; N/A, non-applicable.

**Table 4:** Robust analysis and comparison of treatments across different experimental groups.

Approach	A (n = 59)	B (n = 89)
Characteristic	No pituitary remnants	Adjusted for pituitary remnants
Treatment vs. condition	*p* = 0.021	*p* = 0.002
Interpretation	The effect is biologically authentic	The effect is statistically robust
VTRC-01 vs. rhGH	VTRC-01 vs. rhGH	VTRC-01 vs. rhGH
IC group	*p* = 0.003	*p* = 0.004

**Table 5:** Growth dynamics and potency scaling in the IC group.

Treatment	Growth Rate (mm/Day)	Fold Change in Growth Rate *	*p*
rhGH 5 µg	0.0150	1.0	N/A
VTRC-01 18.0 µg	0.0166	1.1	*p* = 0.509
VTRC-01 20.0 µg	0.0242	1.6	*p* < 0.001

* The fold change in growth rate was calculated by dividing the growth rate of the mRNA-treated groups (18.0 and 20.0 µg) by that of the rhGH-treated group (5 µg).

**Table 6:** Growth dynamics and potency scaling in the Hypox group.

Treatment	Growth Rate (mm/Day)	Fold Change in Growth Rate *	*p*
rhGH 5 µg	0.0082	1.0x	N/A
VTRC-01 8.3 µg	0.0064	0.78x	*p* = 0.314
VTRC-01 12.5 µg	0.0112	1.36x	*p* = 0.106

* The fold change in growth rate was calculated by dividing the growth rate of the mRNA-treated groups (8.3.0 and 12.5.0 µg) by that of the rhGH-treated group (5 µg).

## Data Availability

Dataset available on request from the authors.

## Supplementary Materials

The following supporting information can be downloaded at: https://www.mdpi.com/article/10.3390/cimb48070647/s1.

## Abbreviations

The following abbreviations are used in this manuscript:

%	Percentage
°C	Degrees Celsius
µg	Micrograms
a.u.	Arbitrary units
aa	Amino acids
ANOVA	Analysis of Variance
bp	Base pairs
circRNA	Circular RNA
cm	Centimeters
COVID-19	Coronavirus Disease 2019
CQAs	Critical Quality Attributes
d	Days
DNA	Deoxyribonucleic Acid
e.g.,	Exempli gratia (For example)
FDA	Food and Drug Administration
GHD	Growth Hormone Deficiency
GHRH	Growth Hormone-Releasing Hormone
hGH	Human Growth Hormone
HIV	Human Immunodeficiency Virus
Hypox	Hypophysectomized animal model
IC	Intact Control Group
IGF-1	Insulin-like Growth Factor 1
IM	Intramuscular
ISS	Idiopathic Short Stature
IU	International Units
IVT	In vitro Transcription
kDa	Kilodalton
LiCl	Lithium Chloride
LMM	Linear Mixed Effects Models
LNPs	Lipid Nanoparticles
min	Minutes
mL	Milliliters
mm	Millimeters
mRNA	Messenger Ribonucleic Acid
mV	Millivolts
n	Sample size
nt	Nucleotides
*p*	*p*-value
PDI	Polydispersity Index
rhGH	Recombinant Human Growth Hormone
RNAses	Ribonucleases
SD	Standard Deviation
tLNP	Tissue-specific targeting
TSAs	Tumor-Specific Antigens
UTRs	Untranslated Regions
VTRC-01	Name of product of hGH-mRNA-LNPs
ZP	Zeta Potential
