# Supplementary material for: Design, Synthesis, and Proof-of-Concept Bioassay of an Encapsulated mRNA for Human Growth Hormone
Source: Curr Issues Mol Biol. 2026 Jun 23;48(7):647. doi: 10.3390/cimb48070647 (PMC13406163; doi:10.3390/cimb48070647)
Supplement: Supplementary file 1 [file cimb-48-00647-s001.zip › cimb-4308669-supplementary.pdf]

Table S1 Pilot assays were conducted to optimize the experimental model

Treatment	Experiment 1	Experiment 2	Experiment 3	n
IC	S S S S S	S S S S S S	S S S S S	16
IC + rhGH 5µg			S S S S S S	6
IC +rhGH 200µg		S S S S S		5
IC + mRNA 15µg	S S S S S			5
IC + mRNA 18µg			S S S S	4
IC + mRNA 20µg			S S S S	4
IC + mRNA 34.8µg		S S S S S		5
HYPOX (-)	NS NS S S	PR PR PR S	S S	10
HYPOX + rhGH 5µg		PR PR PR S S	NS NS NS S	9
HYPOX + rhGH 50µg	NS NS PR S			4
HYPOX+ rhGH 200µg		PR PR PR PR S S		6
HYPOX + mRNA 5µg	PR PR S S S			5
HYPOX + mRNA 8.3µg			PR S S S	4
HYPOX + mRNA 12.5µg			NS S S S	4
HYPOX + mRNA 18µg			NS NS NS NS	4
HYPOX + mRNA 20µg			NS NS PR PR	4
HYPOX + mRNA 23µg*	PR NS S			
HYPOX + mRNA 34.8µg		PR PR PR PR PR PR PR		7
HYPOX + mRNA 50µg	NS NS NS NS NS			5
HYPOX + mRNA 100µg	NS NS NS PR S			5
**n**	33	38	41	**Total: 112**

S: Surviving rats   PR: Rats with Pituitary Remnants (PR)   NS: Non-surviving Rats

Table S2 Pilot assays experimental 1

Experiment 1									
Subgroup Description	Initial Pre treatment mesurements			Final post-treatment mesurements			Mesurements at Necropsy		
	Weight (g)	NTBL (cm)	NTL (cm)	Weight (g)	NTBL (cm)	NTL (cm)	Weight (g)	NTBL (cm)	NTL (cm)
Untreated Intact Control	-	-	-	117.0	15.0	29.5	96.6	15.8	30.2
Untreated Intact Control	-	-	-	100.0	14.5	28.9	89.1	14.5	29.7
Untreated Intact Control	-	-	-	124.0	15.3	29.3	98.9	14.5	28.8
Untreated Intact Control	-	-	-	110.5	15.0	28.4	96.0	15.1	29.3
Untreated Intact Control	-	-	-	108.7	15.0	28.5	98.7	15.6	29.7
IC + mRNA 15µg	66.9	13.0	23.9	74.3	12.7	25.1	96.2	14.7	28.3
IC + mRNA 15µg	44.8	11.8	22.0	48.7	11.9	22.7	62.7	13.0	24.5
IC + mRNA 15µg	79.1	13.2	26.0	85.5	14.3	27.8	108.6	15.5	29.5
IC + mRNA 15µg	93.6	14.3	27.4	103.7	14.5	28.2	126.5	15.7	30.2
IC + mRNA 15µg	83.5	13.7	25.7	82.3	13.0	26.0	102.2	14.3	27.5
Untreated Hypox Control	53.9	12.0	23.2	-	17.0	23.7	-	-	-
Untreated Hypox Control	50.6	11.0	21.9	44.7	24.0	22.3	-	-	-
Untreated Hypox Control	58.6	11.5	23.2	60.8	12.0	23.9	62.2	12.2	24.0
Untreated Hypox Control	55.0	11.5	22.6	49.5	14.0	22.3	45.5	12.0	22.6
Hypox + rhGH 50µg	75.3	13.5	26.0	60.1	12.8	24.9	147.2	17.5	32.0
Hypox + rhGH 50µg	50.5	12.0	23.3	48.4	11.7	23.4	-	-	-
Hypox + rhGH 50µg	57.1	12.9	24.3	99.8	15.0	29.1	63.8	12.7	23.7
Hypox + rhGH 50µg	56.1	13.0	24.0	57.0	12.5	24.5	-	-	-
Hypox + mRNA 5µg	60.1	11.5	23.3	65.9	13.0	24.6	73.0	13.3	25.5
Hypox + mRNA 5µg	55.7	12.3	23.9	58.8	11.8	23.3	58.8	13.0	24.7
Hypox + mRNA 5µg	55.3	12.0	23.4	53.2	10.9	22.1	53.4	12.7	23.8
Hypox + mRNA 5µg	68.0	12.7	25.0	89.8	13.5	26.8	104.8	15.0	28.5
Hypox + mRNA 5µg	68.7	12.1	24.0	40.3	11.2	23.0	72.2	13.1	25.7
Hypox + mRNA 50ug*	51.5	12.4	24.3	50.6	11.3	23.6	-	-	-
Hypox + mRNA 50ug	46.4	11.5	22.9	-	-	-	-	-	-
Hypox + mRNA 50ug	51.7	13.0	24.2	-	-	-	-	-	-
Hypox + mRNA 50ug	51.6	12.1	24.5	-	-	-	-	-	-
Hypox + mRNA 50ug	40.1	10.6	21.6	-	-	-	-	-	-
Hypox + mRNA 100ug**	72.6	11.9	24.2	82.3	13.7	27.0	109.4	14.5	28.7
Hypox + mRNA 100ug**	64.0	13.1	25.5	80.8	14.0	27.2	104.0	15.5	29.5
Hypox + mRNA 100ug	60.7	12.3	24.0	-	-	-	-	-	-
Hypox + mRNA 100ug	55.3	13.2	24.5	-	-	-	-	-	-
Hypox + mRNA 100ug	45.1	12.4	22.5	-	-	-	-	-	-

Pituitary Remnants (PR)
NTBL (*Nose-to-Tail Base Length*).
NTL (*Nose-to-Tail Length*).

Table S3 Pilot assays experimental 2

Experiment 2																
Subgroup Description	Pre-Cx	Initial Pre treatment mesurements			Intermediate post-treatment mesurements			Intermediate post-treatment mesurements			Final post-treatment mesurements			Mesurements at End of the experiment		
	Weight (g)	Weight (g)	NTL (cm)	NTBL (cm)	Weight (g)	NTL (cm)	NTBL (cm)	Weight (g)	NTL (cm)	NTBL (cm)	Weight (g)	NTL (cm)	NTBL (cm)	Weight (g)	NTL (cm)	NTBL (cm)
Untreated Intact Control	52.9	130.5	31.0	17.0	161.5	34.7	18.8	158.3	35.3	19.1	166.1	35.4	19.4	170.1	36.0	19.4
Untreated Intact Control	50.3	119.5	29.4	17.2	138.0	33.0	18.0	141.4	33.3	18.1	148.5	33.4	18.5	151.1	34.4	19.0
Untreated Intact Control	58.8	146.0	31.4	17.0	176.7	35.4	19.3	184.0	36.0	19.9	192.3	35.0	19.2	198.0	36.8	20.0
Untreated Intact Control	53.6	109.8	31.2	16.8	143.2	33.5	17.7	144.8	34.0	18.0	145.0	33.8	17.9	151.1	33.8	17.6
Untreated Intact Control	57.3	132.2	31.0	16.6	162.2	34.2	18.5	166.5	34.4	18.4	172.5	35.0	19.2	172.3	35.0	18.7
Untreated Intact Control	49.3	115.4	29.8	16.3	143.4	33.3	18.0	142.9	33.5	18.2	150.2	33.8	18.4	156.3	34.2	18.5
IC +rhGH 200µg	46.3	135.5	30.6	17.1	142.0	33.0	18.1	150.8	33.7	18.7	165.1	34.0	18.7	174.0	34.7	18.5
IC +rhGH 200µg	48.8	134.9	30.5	17.0	148.1	34.0	19.1	158.3	34.3	18.9	174.0	34.4	19.1	184.7	35.4	19.4
IC +rhGH 200µg	48.4	127.4	30.2	16.4	122.3	32.4	17.9	135.7	32.7	18.1	152.7	33.2	18.4	163.2	33.4	18.1
IC +rhGH 200µg	49.9	123.7	29.5	16.0	134.3	32.3	17.5	148.8	33.8	18.5	162.4	33.5	18.4	172.0	35.0	19.4
IC +rhGH 200µg	42.1	120.0	29.8	16.3	126.4	32.2	18.8	141.8	32.2	17.4	153.1	33.0	18.1	168.0	33.7	18.3
IC + mRNA 34.8µg	52.9	130.4	31.9	17.8	145.0	32.9	18.0	149.2	33.4	18.1	169.8	34.0	19.0	184.2	34.9	19.3
IC + mRNA 34.8µg	50.3	99.4	29.6	16.2	164.7	30.2	16.5	105.7	31.0	16.3	125.6	30.9	16.9	136.1	31.9	17.5
IC + mRNA 34.8µg	58.8	103.8	30.5	16.5	113.1	31.0	17.0	120.1	31.0	17.0	133.4	31.5	17.0	141.0	32.4	17.8
IC + mRNA 34.8µg	53.6	116.4	31.2	16.7	131.5	32.5	18.0	140.8	33.2	18.4	158.0	33.7	18.9	168.2	34.3	18.7
IC + mRNA 34.8µg	57.3	116.7	31.9	17.6	133.0	32.0	17.6	134.7	32.2	17.3	150.6	32.9	16.9	158.0	34.0	18.1
Untreated Hypox Control	35.5	42.0	20.5	11.0	40.6	21.0	11.0	40.0	21.4	11.3	43.4	21.6	12.0	43.3	21.0	11.2
Untreated Hypox Control	51.8	62.0	24.4	12.9	61.3	25.3	13.5	63.2	25.7	14.0	65.9	25.7	14.0	67.8	25.6	13.7
Untreated Hypox Control	44.0	47.0	21.3	11.8	43.0	21.1	11.5	44.3	21.7	11.5	49.3	21.7	12.2	53.4	22.0	12.3
Untreated Hypox Control	46.9	51.5	23.2	12.7	50.8	23.6	13.2	51.9	23.5	13.0	54.7	24.2	13.3	52.3	23.8	13.0
Hypox + rhGH 5µg	43.4	56.6	26.9	14.4	108.3	30.0	16.2	108.6	30.4	16.4	113.8	31.2	17.2	118.9	30.3	16.0
Hypox + rhGH 5µg	49.2	50.7	22.9	12.5	62.4	24.7	13.8	63.3	24.7	13.8	66.1	25.1	13.8	63.8	24.5	13.5
Hypox + rhGH 5µg	40.4	68.0	24.1	13.4	86.7	28.0	15.5	91.0	28.4	15.8	93.4	28.9	16.0	100.9	29.3	16.4
Hypox + rhGH 5µg	40.3	57.7	23.0	12.5	65.3	24.6	13.4	62.1	24.9	13.6	68.1	25.2	13.9	68.4	25.0	13.6
Hypox + rhGH 5µg	42.0	64.4	25.0	13.7	76.4	26.4	14.4	73.9	26.7	14.7	79.9	27.0	14.5	74.2	27.2	14.9
Hypox+ rhGH 200µg	38.6	77.8	24.4	13.0	107.8	29.3	15.9	108.2	30.1	16.4	112.9	29.5	15.5	121.1	30.7	16.7
Hypox+ rhGH 200µg	48.0	83.9	27.5	15.0	131.7	30.8	16.5	130.0	31.2	17.0	133.3	31.5	17.5	142.1	31.7	17.4
Hypox+ rhGH 200µg	55.4	63.0	24.8	13.7	82.1	26.5	14.2	78.7	26.5	14.3	80.0	26.8	14.6	82.4	26.8	14.7
Hypox+ rhGH 200µg	52.7	61.5	23.4	12.8	76.5	25.5	14.1	75.2	25.2	14.0	75.5	25.7	14.3	79.1	25.2	13.8
Hypox+ rhGH 200µg	43.8	82.0	26.0	14.4	104.0	29.2	15.8	104.8	30.0	16.3	106.3	29.0	15.9	111.3	29.8	16.2
Hypox+ rhGH 200µg	45.3	80.6	25.9	14.0	129.7	31.0	17.0	120.0	31.0	17.0	125.2	30.7	16.7	126.1	31.3	17.3
Hypox + mRNA 34.8µg	38.6	95.9	28.2	15.5	106.9	31.0	17.8	105.7	31.2	17.0	118.5	30.8	16.7	120.4	31.8	17.0
Hypox + mRNA 34.8µg	41.0	87.5	27.5	15.2	115.1	30.4	16.8	106.6	31.0	16.7	116.3	31.4	17.0	121.2	31.6	17.2
Hypox + mRNA 34.8µg	44.9	93.5	27.8	15.0	118.1	31.6	17.3	116.8	31.5	16.7	126.9	31.8	17.2	127.1	32.2	17.2
Hypox + mRNA 34.8µg	40.6	85.1	26.3	14.0	107.9	30.2	16.4	106.9	30.9	16.7	110.9	31.0	16.7	119.8	31.8	17.2
Hypox + mRNA 34.8µg	49.9	102.6	29.0	12.5	136.5	32.4	18.1	131.5	32.5	18.1	143.5	32.0	17.6	149.1	33.6	18.4
Hypox + mRNA 34.8µg	45.3	99.6	28.8	15.4	123.4	32.5	18.0	123.9	32.0	17.2	135.8	33.3	18.3	144.7	33.9	18.5
Hypox + mRNA 34.8µg	53.5	113.4	29.9	16.2	134.5	33.2	18.2	132.5	34.1	18.4	148.7	34.3	18.5	153.6	34.6	18.4

Pituitary Remnants (PR)
Pre-Cx: Pre-Hypox Surgery baseline measurement
NTBL (*Nose-to-Tail Base Length*).
NTL (*Nose-to-Tail Length*).

Table S4 Pilot assays experimental 3

Experiment 3															
Subgroup Description	Pre-Cx			Initial Pre treatment mesurements			Intermediate post-treatment mesurements			Final post-treatment mesurements			Mesurements at End of the experiment		
	Weight (g)	NTL (cm)	NTBL (cm)	Weight (g)	NTL (cm)	NTBL (cm)	Weight (g)	NTL (cm)	NTBL (cm)	Weight (g)	NTL (cm)	NTBL (cm)	Weight (g)	NTL (cm)	NTBL (cm)
Untreated Intact Control	101.00	27.50	15.40	129.90	31.00	17.30	173.10	34.50	18.80	179.00	35.20	19.20	183.40	35.00	19.20
Untreated Intact Control	70.90	24.30	13.10	98.00	28.30	15.50	147.70	33.10	18.20	154.80	33.50	18.50	167.50	33.90	18.40
Untreated Intact Control	91.30	26.40	14.40	126.70	30.40	16.70	175.40	34.60	19.00	185.00	35.70	20.00	197.30	36.00	19.70
Untreated Intact Control	107.50	28.7	15.7	144.60	32.30	17.90	204.80	36.60	20.10	214.80	36.70	20.10	224.90	37.40	20.00
Untreated Intact Control	98.80	27.80	15.00	134.80	31.30	17.00	167.60	34.50	19.30	177.00	35.50	19.50	194.70	35.70	19.40
IC + rhGH 5µg	99.20	27.60	15.20	127.80	30.40	16.70	196.50	35.40	19.50	214.90	37.00	20.30	225.80	37.30	20.30
IC + rhGH 5µg	80.80	25.70	14.00	122.40	29.60	16.60	195.40	36.30	20.00	203.50	36.50	20.20	217.00	37.40	20.60
IC + rhGH 5µg	89.20	25.70	14.40	126.40	29.50	16.50	183.20	34.10	19.10	191.70	34.90	19.50	205.70	35.30	19.60
IC + rhGH 5µg	104.10	28.00	15.10	137.90	31.50	17.20	197.00	36.20	19.10	208.00	37.40	20.20	216.20	37.60	20.10
IC + rhGH 5µg	105.50	27.50	15.20	136.30	30.50	17.00	203.50	34.90	19.60	211.90	36.10	20.50	219.50	36.50	20.60
IC + rhGH 5µg	92.80	26.90	14.70	128.70	31.30	17.10	182.80	35.00	19.10	194.80	35.80	20.00	207.60	35.70	19.10
IC + mRNA 18µg	90.30	26.20	14.70	124.20	29.40	16.80	179.60	33.90	19.00	186.20	34.80	19.80	200.40	35.40	20.00
IC + mRNA 18µg	93.20	27.60	15.00	120.80	30.50	16.80	162.80	34.40	18.60	169.60	35.10	19.40	180.50	36.30	20.10
IC + mRNA 18µg	94.90	27.00	14.80	130.40	31.00	16.70	179.20	34.70	18.50	180.30	36.60	19.50	197.90	36.70	19.90
IC + mRNA 18µg	92.80	27.00	14.70	120.50	30.80	17.00	162.60	35.50	19.10	172.00	35.80	19.20	178.10	35.80	19.50
IC + mRNA 20µg	77.30	25.10	13.60	116.60	29.10	16.00	187.10	36.00	19.30	200.70	36.80	20.10	216.50	37.90	21.20
IC + mRNA 20µg	92.80	27.00	14.70	133.70	30.70	17.00	191.40	36.00	19.50	199.30	36.50	19.80	215.00	37.50	20.40
IC + mRNA 20µg	90.00	26.70	14.70	121.60	29.90	16.50	176.20	34.20	18.60	183.90	35.50	19.50	213.30	35.80	19.50
IC + mRNA 20µg	87.20	26.60	14.60	122.00	30.40	16.70	172.50	35.00	19.30	186.00	36.50	20.20	208.30	37.20	20.60
Untreated Hypox Control	72.90	25.80	13.90	75.00	26.10	15.50	73.00	26.20	14.30	69.30	26.20	14.30	73.80	26.20	14.10
Untreated Hypox Control	68.20	25.70	14.20	71.20	26.30	14.50	67.50	26.10	14.20	65.70	26.10	14.20	64.20	26.40	13.90
Hypox + rhGH 5µg	73.90	25.10	14.20	71.00	25.30	14.00	84.30	27.00	15.00	91.80	27.40	15.00	90.70	27.50	15.00
Hypox + rhGH 5µg	-	-	-	-	-	-	-	-	-	-	-	-	-	-	-
Hypox + rhGH 5µg	-	-	-	-	-	-	-	-	-	-	-	-	-	-	-
Hypox+mRNA(8.3)	68.00	25.00	13.10	66.30	25.80	14.00	57.70	25.60	14.00	56.50	25.60	14.00	59.60	26.00	14.00
Hypox+mRNA(8.3)	69.30	24.90	13.00	66.00	25.90	13.80	73.30	26.50	14.10	73.60	26.50	14.30	73.00	26.30	14.20
Hypox+mRNA(8.3)	53.00	24.00	13.00	54.50	24.00	13.00	54.10	24.60	13.40	49.20	24.60	13.40	42.90	25.20	13.60
Hypox+mRNA(8.3)	64.40	24.60	13.30	59.80	25.60	13.60	65.50	25.50	14.00	72.00	25.50	14.00	71.40	26.30	14.40
Hypox+mRNA(12.5)	56.80	23.70	12.60	61.50	24.30	13.30	63.20	25.20	13.90	63.20	25.20	13.90	65.00	25.20	13.70
Hypox+mRNA(12.5)	59.40	24.20	13.20	54.80	24.70	13.50	68.00	25.90	14.60	68.90	25.90	14.70	61.80	25.90	14.20
Hypox+mRNA(12.5)	50.50	23.20	12.80	54.70	23.40	12.80	61.10	24.50	13.80	66.00	24.50	13.80	67.70	25.20	13.70
Hypox+mRNA(12.5)	-	-	-	-	-	-	-	-	-	-	-	-	-	-	-
Hypox + mRNA 18µg	-	-	-	-	-	-	-	-	-	-	-	-	-	-	-
Hypox + mRNA 18µg	-	-	-	-	-	-	-	-	-	-	-	-	-	-	-
Hypox + mRNA 18µg	-	-	-	-	-	-	-	-	-	-	-	-	-	-	-
Hypox + mRNA 18µg	-	-	-	-	-	-	-	-	-	-	-	-	-	-	-
Hypox+mRNA(20/18)	50.70	24.00	13.00	72.40	25.70	14.00	111.30	29.70	15.60	115.90	31.20	17.10	130.80	32.00	17.20
Hypox+mRNA(20/18)	54.60	23.50	13.00	55.60	23.60	13.20	96.80	27.60	15.30	98.30	28.00	15.70	107.70	28.40	15.40
Hypox+mRNA(20/18)	-	-	-	-	-	-	-	-	-	-	-	-	-	-	-
Hypox+mRNA(20/18)	-	-	-	-	-	-	-	-	-	-	-	-	-	-	-

Pituitary Remnants (PR)
Pre-Cx: Pre-Hypox Surgery baseline measurement
NTBL (*Nose-to-Tail Base Length*).
NTL (*Nose-to-Tail Length*).
